# Seven-year outcomes following intensive anti-vascular endothelial growth factor therapy in patients with exudative age-related macular degeneration

**DOI:** 10.1186/s12886-023-02843-2

**Published:** 2023-03-17

**Authors:** Regina Lukacs, Miklos Schneider, Zoltan Zsolt Nagy, Gabor Laszlo Sandor, Kinga Kaan, Antonia Asztalos, Lajos Enyedi, Gyorgy Pek, Gyorgy Barcsay, Antal Szabo, Agnes Borbandy, Illes Kovacs, Miklos Denes Resch, Andras Papp

**Affiliations:** 1 Department of Ophthalmology, Flor Ferenc Hospital of Pest County, Kistarcsa, Hungary; 2 Department of Ophthalmology, Semmelweis University, Maria u. 39, Budapest, H-1085 Hungary; 3 Department of Ophthalmology, Rigshospitalet, Glostrup, Denmark; 4 Department of Ophthalmology, Bajcsy-Zsilinszky Hospital, Budapest, Hungary

**Keywords:** Exudative, Wet, Age-related macular degeneration, AMD, Retina, Macula, Aflibercept, Ranibizumab, Anti-VEGF, Long-term treatment

## Abstract

**Background:**

Anti-vascular endothelial growth factor (VEGF) therapy is currently the most effective therapy of exudative age-related macular degeneration (AMD). The aim of this study was to assess long-term benefits of intensive aflibercept and ranibizumab anti-VEGF therapy in patients with exudative AMD.

**Methods:**

Two clinical trial sites recruited their original subjects for a re-evaluation 7 years after the baseline visit of the phase-3 Vascular Endothelial Growth Factor (VEGF) Trap-Eye: Investigation of Efficacy and Safety in Wet Age-Related Macular Degeneration (VIEW 2) trial.

Forty-seven eyes of 47 patients with AMD originally treated with ranibizumab (14 eyes) or aflibercept (33 eyes) were included.

**Results:**

Mean number of injections was 17.8 ± 3.0 during participation in the VIEW 2 trial. Fourteen of 47 (30%) eyes were given additional injections with a mean number of 5.7 ± 4.5 after the trial. At a mean follow-up time of 82 ± 5 months best corrected visual acuity (BCVA) remained stable or improved (≤ 10 letters lost) in 55% of patients in the entire study population, in 43% in the ranibizumab group and in 60% in the aflibercept group. In both groups combined mean BCVA was 54 ± 13 letters at baseline, 65 ± 17 letters at the end of the intensive phase and 45 ± 25 letters at the end of follow-up. There was no statistically significant difference in BCVA between the two groups at baseline (*p* = 0.88) and at the end of follow-up (*p* = 0.40). Macular atrophy was observed in 96% of eyes, average area was 7.22 ± 6.31 mm^2^ with no statistically significant difference between groups (*p* = 0.47). Correlation between BCVA at end-of-follow-up and the area of atrophy was significant (*p* < 0.001). At the end of follow-up, fluid was detected in 7 of 47 eyes (15%) indicating disease activity.

**Conclusion:**

Long-term efficacy of aflibercept and ranibizumab was largely consistent. Following a two-year intensive therapy with as-needed regimen, BCVA was maintained or improved in almost half of the patients and in the ranibizumab group and more than half of the patients in the aflibercept group with very few injections. In a remarkable proportion of eyes, BCVA declined severely which underlines the need for long-term follow-ups and may indicate a more prolonged intensive therapy.

**Trial registrations:**

VIEW 2 study: ClinicalTrials.gov ID: NCT00637377, date of registration: March 18, 2008.

Long-term follow-up: IRB nr.: SE RKEB 168/2022, ClinicalTrials.gov ID: NCT05678517, date of registration: December 28, 2022, retrospectively registered.

## Background

Anti-vascular endothelial growth factor (anti-VEGF) therapy has been the most effective therapy of exudative (wet) age-related macular degeneration (AMD) for over a decade and is currently considered standard-of-care. Aflibercept has a more prolonged and potent anti-VEGF effect compared to prior treatments (bevacizumab and ranibizumab) [1]. The reason for this enhanced effect is that aflibercept binds to VEGF-B and placental growth factor (PGF) isoforms as well in addition to isoforms of VEGF-A, whereas bevacizumab and ranibizumab only binds to the latter [2]. Additionally, compared to ranibizumab, aflibercept has a longer intravitreal half-life that allows a less frequent treatment regimen [2–4]. Aflibercept has also been proven to be effective in other diseases such as diabetic macular edema [5] and central retinal vein occlusion [6, 7] where VEGF plays an important role in the pathogenesis.

Long-term outcomes of anti-VEGF treatments on wet AMD are less frequently documented in clinical trials as the length of those are usually limited to one to two years. However, in the recent past, several papers, many of which based on real-life data, were published presenting the long-term efficacy of intravitreal anti-VEGF agents and the number of given injections with variable results [8–22].

This study aims to assess long-term benefits of intensive anti-VEGF therapy followed by a very low number of injections in patients with exudative AMD.

## Methods

### Study design and patient recruitment

This study was conducted at the Department of Ophthalmology at Semmelweis University and the Bajcsy-Zsilinszky Hospital in Budapest, Hungary. Study participants were patients with neovascular AMD, subjects of two sites in Budapest, Hungary of the “Vascular Endothelial Growth Factor (VEGF) Trap-Eye: Investigation of Efficacy and Safety in Wet Age-Related Macular Degeneration (VIEW 2)” phase-3 multicenter, prospective, randomized, double blind clinical trial (ClinicalTrials.gov ID: NCT00637377) [23]. The trial was carried out in accordance with the tenets of the Declaration of Helsinki, with approval of respective institutional review boards of the participating centers. For the long-term follow-up after the VIEW 2 study, institutional review board approval was obtained for a retrospective analysis (IRB approval nr: SE RKB 168/2022, ClinicalTrials.gov ID: NCT05678517). Written informed consent was obtained from each participant before enrollment and at the end of follow-up for the retrospective analysis.

Patients were treated with intravitreal anti-VEGF injections between 2008 and 2017. During the first 2 years in the framework of the VIEW 2 study (intensive phase), participants were randomized per protocol to intravitreal aflibercept (aflibercept group) or ranibizumab treatment arms (ranibizumab group). In the first year, patients received aflibercept injections every 4 or 8 weeks or ranibizumab every 4 weeks. During the second year, patients received injections every 4–12 weeks with a minimum of dosing every 12 weeks and interim as-needed monthly injections.

After finishing the intensive phase, patients were followed under regular clinical care in real-life conditions (post-intensive phase) and both groups were treated with predominantly ranibizumab anti-VEGF injections (3 patients received aflibercept occasionally when ranibizumab was not available). Treatment was administered *pro re nata* (PRN, as needed). Retreatment criteria in the post-intensive phase were based on visual acuity (more than 5 letters worsening from the previous visit), signs of activity on optical coherence tomography (OCT) (+ 50 µm increase in central retinal thickness from the previous visit) or indirect slit lamp biomicroscopy (new hemorrhage at the lesion). The decision to retreat ultimately rested on the judgement of the patient’s treating physician, taking the availability of the medicine into account.

Results at the end of the follow-up were retrospectively analyzed.

### Study objectives

Primary outcomes of the study were change in best corrected visual acuity (BCVA), change in size of geographic atrophy on fundus autofluorescence (FAF) and presence or absence of intra- or subretinal fluid on spectral-domain optical coherence tomography (OCT) at the end of follow-up. BCVA was measured with standardized Early Treatment Diabetic Retinopathy Study (ETDRS) visual acuity charts. Visual acuity was defined as stable when less than 10 letters were gained or lost, improved when at least 10 letters were gained and worsened when at least 10 letters were lost on the ETDRS chart. FAF and OCT examinations were performed on the Spectralis OCT (Heidelberg Engineering GmbH, Heidelberg, Germany) device. Area of geographic atrophy was calculated using the built-in software (Heidelberg Eye Explorer, version: 1.10.2.0) of the Spectralis OCT device following manual marking.

Secondary endpoints included number of injections and potential adverse events.

### Statistical analysis

Mann–Whitney test was used to compare differences in mean visual acuity and geographic atrophy size between the start and end of follow-up in the two treatment groups. Wilcoxon test was used to assess changes in visual acuity in groups together and separately. The correlation between BCVA at the end of follow-up and the area of atrophy was evaluated with Spearman test. For rounding percentage values, the largest remainder method was used.

Statistica 8.0 (Statsof, Tulsa OK, USA) software was used for the analysis. Statistically significant difference was considered as *p* < 0.05.

## Results

### Patient characteristics and follow-up time

Forty-seven eyes of 47 patients were included in this study. Among the participants there were 16 men and 31 women. Average age ± standard deviation of the study patients was 71 ± 8 [range: 53–84] years. Mean follow-up time was 82 ± 5 months.

### Number of injections

Fourteen of the 47 eyes received intravitreal ranibizumab and 33 eyes received intravitreal aflibercept injections. Mean number of injections was 17.8 ± 3.0 during the intensive phase and 19.5 ± 5.0 during the entire follow-up. During the post-intensive phase, 14 of 47 eyes (30%) were given additional injections with a mean number of 5.7 ± 4.5 [minimum: 1, maximum: 13] per patient, while the remaining 70% received no injections. Of those who did not receive injections during the post-intensive phase, 8 patients (17%) had lasting stability with no need for treatment, 13 patients (28%) had poor visual prognosis due to fibrosis, atrophy or subretinal bleeding, and the remaining 12 (25%) did not receive injections due to compliance problems or budgetary restrictions.

### Visual acuity

**Fig. 1:**
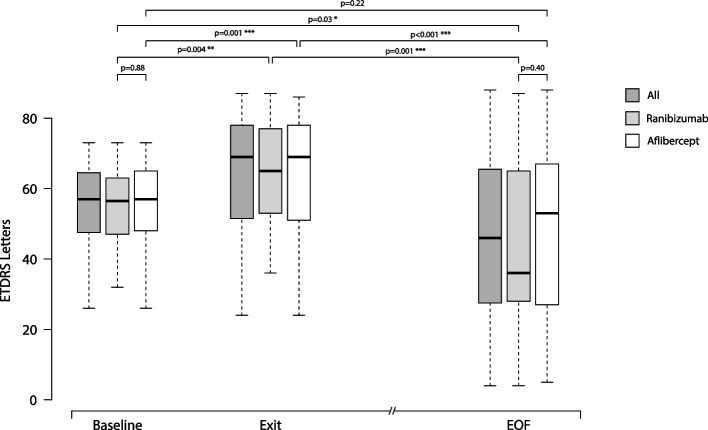
Box and Whisker plot graph showing changes in best corrected visual acuity during follow-up: baseline, exit of study and end of follow-up. Abbreviation: BCVA: Best corrected visual acuity. Single asterisk (*) indicates *p* ≤ 0.05, double asterisk (**) indicates *p* ≤ 0.01, triple asterisk (***) indicates *p* ≤ 0.001. Abbreviation: EOF: end of follow-up

**Fig. 2:**
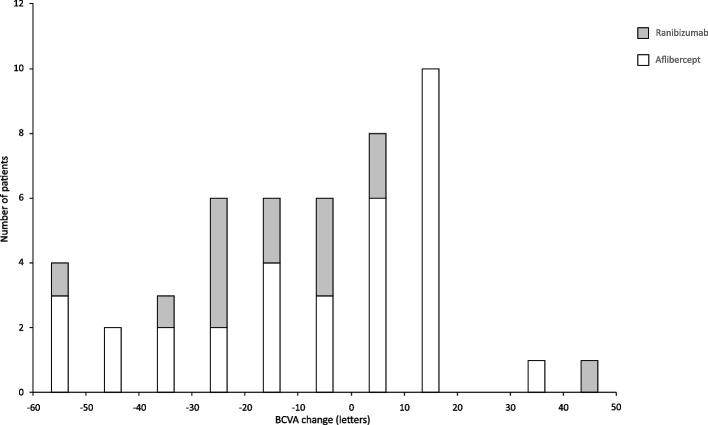
Bar graph showing distribution of eyes grouped by number of lost or gained ETDRS letters at the end of follow-up. Abbreviation: BCVA: Best corrected visual acuity

In both groups combined, mean BCVA was 54 ± 13 letters at baseline, 65 ± 17 letters at the end of the intensive phase and 45 ± 25 letters at the end of follow-up (Fig. 1). Visual acuity at the end of follow-up was stable in 14 of 47 eyes (30%), improved in 12 eyes (25%) and worsened in 21 eyes (45%) (Fig. 2).

Difference in visual acuity in all eyes at the end of follow-up was statistically significant compared to baseline values (*p* = 0.02). Mean change in ETDRS letters was statistically significant at the end of the intensive phase compared to baseline (+ 10 ± 14 letters, *p* < 0.001), and at the end of follow-up compared to the end of intensive phase (-19 ± 23 letters, *p* < 0.001).

In the ranibizumab group, mean BCVA score was 54 ± 13 letters at baseline, 64 ± 18 letters at the end of intensive phase, and 41 ± 25 letters at the end of follow-up (Fig. 1). At the end of follow-up 5 of 14 eyes (36%) had stable visual acuity, 1 eye (7%) improved and 8 eyes (57%) got worse (Fig. 2).

In the aflibercept group, mean BCVA was 55 ± 13 letters at baseline, 65 ± 17 letters at the end of intensive phase, then decreased to 47 ± 25 letters by the end of follow-up (Fig. 1). At the end of follow-up 11 of 33 eyes (33%) had stable visual acuity, 9 eyes (27%) improved, and 13 eyes (40%) worsened (Fig. 2).

Statistical results in separate groups and the total study population were largely consistent. Visual acuity changes were significant at the end of intensive phase compared to baseline (*p* = 0.004 and *p* = 0.001 in the ranibizumab and aflibercept-treated group, respectively) and at the end of follow-up compared to the end of intensive phase (*p* = 0.001 and *p* < 0.001 in the ranibizumab and aflibercept-treated group, respectively). Visual acuity decrease at the end of follow-up was significant compared to baseline values in the ranibizumab (*p* = 0.03) but was not significant in the aflibercept-treated group (*p* = 0.22). There was no statistically significant difference in BCVA between the two treatment groups at baseline (*p* = 0.88) and at the end of follow-up (*p* = 0.40) (Fig. 1).

### Area of atrophy and disease activity

**Fig. 3:**
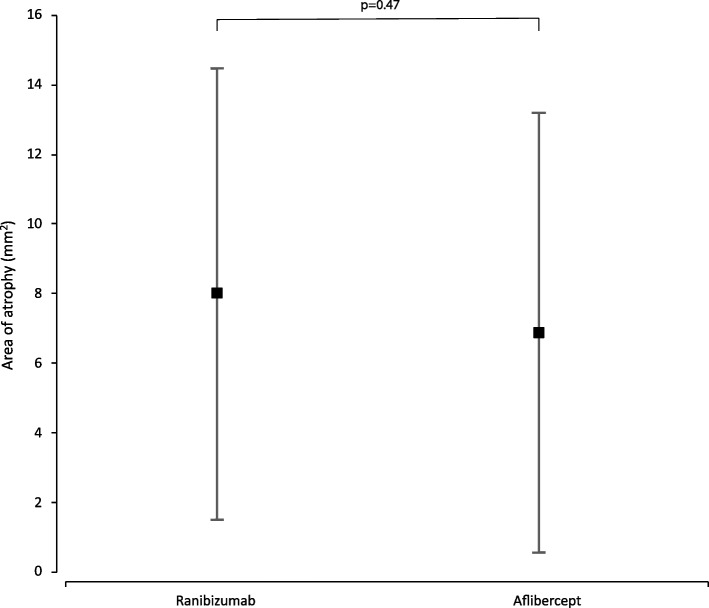
Graph showing difference in area of macular atrophy (mean ± SD) between ranibizumab and aflibercept treatment groups at the end of follow-up. Difference between groups was statistically not significant (*p* = 0,4708)

**Fig. 4:**
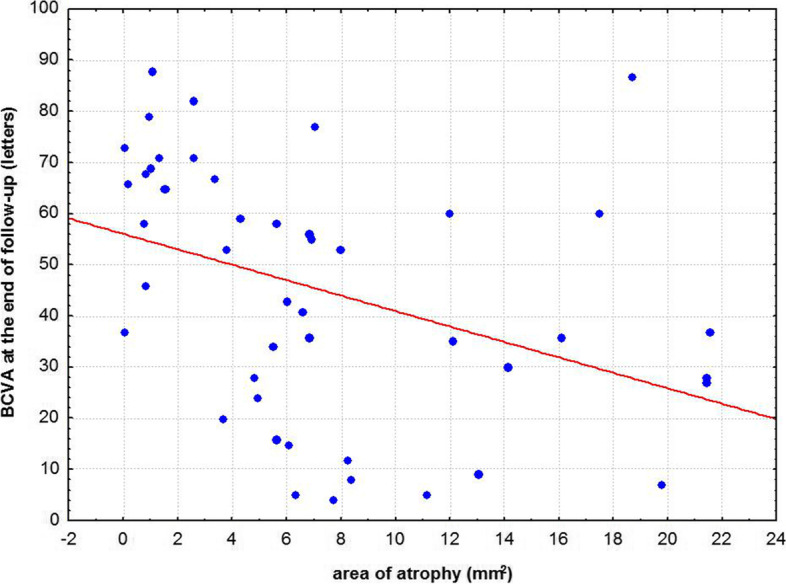
Graph showing the relationship between best corrected visual acuity and area of macular atrophy at the end of follow-up. Correlation was statistically significant (*p* = 0,0002)

At the end of follow-up, we observed macular atrophy in 96% of the study eyes. Average area of macular atrophy measured on FAF was 7.22 ± 6.31 mm^2^. Average area of atrophy was 8.00 ± 6.48 mm^2^ in the ranibizumab group and 6.89 ± 6.31 mm^2^ in the aflibercept group. Difference between the treatment groups was not statistically significant (*p* = 0.47) (Fig. 3). The correlation between BCVA at the end of follow-up and the area of atrophy was statistically significant (Spearman *R* = -0.50, *p* < 0.001) (Fig. 4).

At the end of follow-up, fluid was detected on OCT images in 7 of 47 eyes (15%) indicating disease activity, 3 of which (6%) were patients not receiving treatment in the post-intensive phase.

### Adverse events

During follow-up, we observed no serious ocular adverse events. Eight of 47 eyes (17%) underwent cataract surgery during the trial. Serious systemic adverse events included stroke, which occurred in two patients during the entire follow-up period.

## Discussion

Short-term benefits of aflibercept and ranibizumab treatment in exudative AMD were extensively published previously [24–28]. In the recent past, some clinical trials had extended follow-up periods [8, 10, 12, 16, 29], a handful papers with long-term real-life data [9, 13, 15, 17, 18, 21, 30–32] and a few systematic reviews of long-term results were published [14, 19].

Our study reports on the 7-year long-term outcomes of a 2-year-long intensive anti-VEGF treatment followed by a 5-year period with a very low number of injections and compares long-term results between groups initially treated with aflibercept or ranibizumab.

In the first two years, our patients were included in the VIEW 2 clinical trial. The VIEW 1 and VIEW 2 clinical trials were two multicenter, phase-3, randomized studies aiming to compare monthly or bimonthly administered intravitreal aflibercept, with monthly administered ranibizumab in patients with exudative AMD [23]. At week 52, proportion of patients losing < 15 letters on ETDRS chart was similar in all treatment groups ranging from 94.4% to 96.1%. Anatomic improvements were also comparable between treatment groups. The studies concluded that aflibercept treatment given monthly or bimonthly after 3 initial monthly injections was proven noninferior to monthly ranibizumab. Additionally, occurrence of ocular or systematic adverse events in aflibercept groups was not more frequent. In the second year of the VIEW 1 and 2 studies the observed anatomic and visual improvement was maintained using an as-needed regimen with fixed quarterly dosing [33]. The proportion of patients maintaining BCVA was 91.5% to 92.4%, results were similar between treatment groups and aflibercept was noninferior to ranibizumab treatment at week 96.

Results of our 47 patients at the end of year 2 were similar to those of the VIEW 1 and 2 studies, which is not surprising since they were treated according to the same protocol. Visual acuity of our patients improved significantly, mean gain was 11 ETDRS letters achieved by an average of 18 injections. In the VIEW 1 and 2 studies an average improvement of 6.6 to 7.9 letters was seen at week 96 requiring 11.2 to 16.5 injections [33]. In the separate treatment groups results were similar, both ranibizumab and aflibercept treatment resulted in significant visual gain by the end of the second year, showing that aflibercept and ranibizumab treatments were equally effective.

A retrospective study by Eleftheriadou et al. analyzing 2-year treatment results of aflibercept in neovascular AMD had comparable promising results. With a mean of 11.4 injections they observed an average 5.1-letter improvement [34]. Similarly to the former ones, most clinical trials and real-life studies agree on the fact that short-term results of intravitreal anti-VEGF treatment are very positive [10, 11, 21].

A relevant question remains: can this favorable effect be maintained on the long term? In our study we tried to find an answer to that question by observing our patients for an average of 82 months. At the end of our follow-up, mean visual acuity declined in the entire study population compared with both baseline and end-of-intensive-phase values. Our results were similar in the ranibizumab and aflibercept groups. Comparing visual acuity at end of follow-up with the end of intensive phase, the decline was more remarkable and statistically significant, mean decrease was 20 letters in all groups together and results were similar in both treatment groups. Our results are comparable with the SEVEN-UP study [8] which aimed to assess long-term results of ranibizumab treatment in patients with exudative AMD. The SEVEN-UP study included patients who completed 24 months in the ANCHOR or the MARINA trial and then also completed another 24 months in the HORIZON study in the ranibizumab arm. Seven years after the initial entry, patients were called back for a single reassessment visit, when a mean decrease of 8.6 letters (*p* < 0.005) was observed from baseline despite continuous treatment in years 2–7 [8].

After the second year, the number of injections given to our patients decreased remarkably. Similarly, in the SEVEN-UP study the number of injections given notably decreased after the exit of the initial ANCHOR or MARINA and HORIZON trials. During the period between the HORIZON exit and end of follow-up (3.4 years on average) mean number of injections given was 6.8 per eye, yet it meant far more treatments per eye compared to our trial (5.7 injections on average during the 4.8 years of the post-intensive phase in those treated, while 70% of our patients did not receive treatment). Such a low number of injections can potentially explain the long-term vision decrease in ranibizumab and aflibercept-treated patients. In a study with a 10-year follow-up Garweg et al. had similar results, 7.3 to 11.9 letters of visual decline in years 3–10 with a mean of 2.8 yearly injections [13]. A meta-analysis by Gerding concluded that the cumulative number of injections correlates to the gained and maintained visual improvement [14].

At the end of follow-up, macular atrophy was observed in 96% of our patients, and the area of atrophy was associated with decreased visual acuity, their relationship was statistically significant. Similarly, in the SEVEN-UP study 98% of eyes had atrophy, the average area of atrophy was 9.4 ± 7.4 mm^2^, correlation between declined BCVA and the extent of macular atrophy was statistically significant [8]. Despite the treatment, macular atrophy developed in most patients eventually which can be another potential explanation for the long-term visual acuity decline [8]. In the second report of the SEVEN-UP study, authors concluded that macular atrophy had a growth rate of 0.28 mm.^2^/year, its progression was significantly associated with visual decline and confirmed that final macular atrophy size was significantly related to final visual acuity [22]. Another study by Munk et al. focused on the prevalence and progression of macular atrophy and found that the majority (73.5%) of their patients showed atrophy after long-term anti-VEGF treatment [17]. Berg et al. also concluded that macular atrophic development is the most likely explanation for long-term visual decline [11].

The SEVEN-UP study also showed that disease activity can be observed even long-term in a considerable proportion of patients (exudation was proven by OCT evidence in 68% of the eyes examined) [8]. At the end of our trial we observed disease activity in only 15% of eyes. These data highlight that patients with exudative AMD need long-term follow-up and treatment can be necessary even after several years of disease detection.

We studied the proportion of patients with maintained or improved visual acuity during follow-up as well. BCVA remained stable or improved in 55% of patients in the entire study population, in 43% in the ranibizumab group and in 60% in the aflibercept group.

We observed long-term vision improvement in a remarkable proportion of patients, especially in the aflibercept group despite the very low number of injections after the second year. In these patients, visual improvement could be maintained even after the two years of intensive treatment for a prolonged period with only a few injections or without any. In the SEVEN-UP study, results are similar, 43% of eyes had stable or improved BCVA compared with baseline values [8]. However, the other portion of our patients experienced visual decline, 45% in the whole patient pool, 57% in the ranibizumab group and 39% in the aflibercept group.

In another trial, Peden et al. [18] also studied the long-term effects of anti-VEGF (primarily ranibizumab) therapy in exudative AMD for at least 5 years. In contrast to the SEVEN-UP, in this study patients were treated with fixed-interval injections administered in every 4 to 8 weeks. At year 7, mean visual improvement was 12.1 letters, a vastly different result than that of the SEVEN-UP study and what we observed in our trial. These outcomes support the superiority of long-term fixed-interval intensive treatment in exudative AMD.

Another interesting observation regarding long-term treatment by Sagiv et al. was that good final visual acuity was associated with good initial visual acuity, highlighting the benefits of early detection and treatment [20].

Our study has several limitations, first being its retrospective nature, second, its low number of participants, and third, the non-standardized treatment regimen in the post-intensive phase. Patients after exiting the VIEW 2 study returned to real-life conditions and were treated by their physicians on an as-needed basis, which was sometimes severely limited by budgetary constraints resulting in undertreatments. Additionally, patient compliance got worse over the course of the follow-up, not all patients showed up for their check-ups or treatment sessions. After the VIEW 2 study, patients could not continue receiving their original trial medication, since at that time the treatment of choice was ranibizumab, aflibercept was only used later when ranibizumab was not available. This happened in a total of 3 patients on 3, 5 and 5 occasions, respectively.

In conclusion, long-term efficacy of aflibercept and ranibizumab was similar in our patients with exudative AMD. During our nearly 7 year-long follow-up, both drugs proved safe and well tolerated with no serious ocular adverse events. Following a two-year intensive therapy with an as needed regimen, visual acuity was maintained or improved in almost half of the patients in the ranibizumab group, and more than half of the patients in the aflibercept group, with very few injections. In a remarkable proportion of eyes, visual acuity declined severely during the post-intensive period which underlines the need for long-term follow-ups and may indicate a more prolonged intensive therapy.

## Data Availability

The datasets used and/or analyzed during the current study are available from the corresponding author on reasonable request.

## Abbreviations

AMD: Age-related macular degeneration
ANCHOR: Anti-VEGF Antibody for the Treatment of Predominantly Classic Choroidal Neovascularization in Age-Related Macular Degeneration
BCVA: Best corrected visual acuity
ETDRS: Early Treatment Diabetic Retinopathy Study
FAF: Fundus autofluorescence
HORIZON: An Open-Label Extension Trial of Ranibizumab for Choroidal Neovascularization Secondary to Age-Related Macular Degeneration
MARINA: Minimally Classic/Occult Trial of the Anti-VEGF Antibody Ranibizumab in the Treatment of Neovascular Age-Related Macular Degeneration
OCT: Optical coherence tomography
SD: Standard deviation
VEGF: Vascular endothelial growth factor
VIEW 2: Vascular Endothelial Growth Factor (VEGF) Trap-Eye: Investigation of Efficacy and Safety in Wet Age-Related Macular Degeneration (VIEW 2) trial

## References

[1] Stewart MW (2012). Clinical and differential utility of VEGF inhibitors in wet age-related macular degeneration: focus on aflibercept. Clin Ophthalmol.

[2] Papadopoulos N, Martin J, Ruan Q, Rafique A, Rosconi MP, Shi E (2012). Binding and neutralization of vascular endothelial growth factor (VEGF) and related ligands by VEGF Trap, ranibizumab and bevacizumab. Angiogenesis.

[3] Garcia-Layana A, Figueroa MS, Araiz J, Ruiz-Moreno JM, Gomez-Ulla F, Arias-Barquet L (2015). Treatment of exudative age-related macular degeneration: focus on aflibercept. Drugs Aging.

[4] Semeraro F, Morescalchi F, Duse S, Parmeggiani F, Gambicorti E, Costagliola C (2013). Aflibercept in wet AMD: specific role and optimal use. Drug Des Devel Ther.

[5] Do DV, Schmidt-Erfurth U, Gonzalez VH, Gordon CM, Tolentino M, Berliner AJ (2011). The DA VINCI study: phase 2 primary results of VEGF trap-eye in patients with diabetic macular edema. Ophthalmology.

[6] Brown DM, Heier JS, Clark WL, Boyer DS, Vitti R, Berliner AJ (2013). Intravitreal aflibercept injection for macular edema secondary to central retinal vein occlusion: 1-year results from the phase 3 COPERNICUS study. Am J Ophthalmol.

[7] Holz FG, Roider J, Ogura Y, Korobelnik JF, Simader C, Groetzbach G (2013). VEGF trap-eye for macular oedema secondary to central retinal vein occlusion: 6-month results of the phase III GALILEO study. Br J Ophthalmol.

[8] Rofagha S, Bhisitkul RB, Boyer DS, Sadda SR, Zhang K, Group S-US (2013). Seven-year outcomes in ranibizumab-treated patients in ANCHOR, MARINA, and HORIZON: a multicenter cohort study (SEVEN-UP). Ophthalmology.

[9] Adrean SD, Chaili S, Ramkumar H, Pirouz A, Grant S (2018). Consistent long-term therapy of neovascular age-related macular degeneration managed by 50 or more anti-VEGF injections using a treat-extend-stop protocol. Ophthalmology.

[10] Amstutz CA, Fleischhauer J, Zweifel S, Barthelmes D (2015). Long-term outcome in patients with intravitreal anti-VEGF Therapy for Exudative AMD. Klin Monbl Augenheilkd.

[11] Berg K, Roald AB, Navaratnam J, Bragadottir R (2017). An 8-year follow-up of anti-vascular endothelial growth factor treatment with a treat-and-extend modality for neovascular age-related macular degeneration. Acta Ophthalmol.

[12] Maguire MG, Martin DF, Ying GS, Jaffe GJ, Daniel E, Comparison of Age-related Macular Degeneration Treatments Trials Research G (2016). Five-year outcomes with anti-vascular endothelial growth factor treatment of neovascular age-related macular degeneration: the comparison of age-related macular degeneration treatments trials. Ophthalmology.

[13] Garweg JG, Zirpel JJ, Gerhardt C, Pfister IB (2018). The fate of eyes with wet AMD beyond four years of anti-VEGF therapy. Graefes Arch Clin Exp Ophthalmol.

[14] Gerding H (2016). Long-term results of intravitreal anti-VEGF injections in wet AMD: a meta-analysis. Klin Monbl Augenheilkd.

[15] Gillies MC, Campain A, Barthelmes D, Simpson JM, Arnold JJ, Guymer RH (2015). Long-term outcomes of treatment of neovascular age-related macular degeneration: data from an observational study. Ophthalmology.

[16] Keenan TD, Vitale S, Agron E, Domalpally A, Antoszyk AN, Elman MJ (2020). Visual acuity outcomes after anti-vascular endothelial growth factor treatment for neovascular age-related macular degeneration: age-related eye disease study 2 report number 19. Ophthalmol Retina.

[17] Munk MR, Ceklic L, Ebneter A, Huf W, Wolf S, Zinkernagel MS (2016). Macular atrophy in patients with long-term anti-VEGF treatment for neovascular age-related macular degeneration. Acta Ophthalmol.

[18] Peden MC, Suner IJ, Hammer ME, Grizzard WS (2015). Long-term outcomes in eyes receiving fixed-interval dosing of anti-vascular endothelial growth factor agents for wet age-related macular degeneration. Ophthalmology.

[19] Rasmussen A, Sander B (2014). Long-term longitudinal study of patients treated with ranibizumab for neovascular age-related macular degeneration. Curr Opin Ophthalmol.

[20] Sagiv O, Zloto O, Moroz I, Moisseiev J (2017). Different clinical courses on long-term follow-up of age-related macular degeneration patients treated with intravitreal anti-vascular endothelial growth factor injections. Ophthalmologica.

[21] Wecker T, Ehlken C, Buhler A, Lange C, Agostini H, Bohringer D (2017). Five-year visual acuity outcomes and injection patterns in patients with pro-re-nata treatments for AMD, DME, RVO and myopic CNV. Br J Ophthalmol.

[22] Bhisitkul RB, Mendes TS, Rofagha S, Enanoria W, Boyer DS, Sadda SR (2015). Macular atrophy progression and 7-year vision outcomes in subjects from the ANCHOR, MARINA, and HORIZON studies: the SEVEN-UP study. Am J Ophthalmol.

[23] Heier JS, Brown DM, Chong V, Korobelnik JF, Kaiser PK, Nguyen QD (2012). Intravitreal aflibercept (VEGF trap-eye) in wet age-related macular degeneration. Ophthalmology.

[24] Brown DM, Kaiser PK, Michels M, Soubrane G, Heier JS, Kim RY (2006). Ranibizumab versus verteporfin for neovascular age-related macular degeneration. N Engl J Med.

[25] Brown DM, Michels M, Kaiser PK, Heier JS, Sy JP, Ianchulev T (2009). Ranibizumab versus verteporfin photodynamic therapy for neovascular age-related macular degeneration: two-year results of the ANCHOR study. Ophthalmology.

[26] Heier JS, Boyer D, Nguyen QD, Marcus D, Roth DB, Yancopoulos G (2011). The 1-year results of CLEAR-IT 2, a phase 2 study of vascular endothelial growth factor trap-eye dosed as-needed after 12-week fixed dosing. Ophthalmology.

[27] Nguyen QD, Shah SM, Browning DJ, Hudson H, Sonkin P, Hariprasad SM (2009). A phase I study of intravitreal vascular endothelial growth factor trap-eye in patients with neovascular age-related macular degeneration. Ophthalmology.

[28] Rosenfeld PJ, Brown DM, Heier JS, Boyer DS, Kaiser PK, Chung CY (2006). Ranibizumab for neovascular age-related macular degeneration. N Engl J Med.

[29] Evans RN, Reeves BC, Phillips D, Muldrew KA, Rogers C, Harding SP (2020). Long-term visual outcomes after release from protocol in patients who participated in the Inhibition of VEGF in Age-related Choroidal Neovascularisation (IVAN) Trial. Ophthalmology.

[30] Arpa C, Khalid H, Chandra S, Wagner S, Fasler K, Faes L, et al. Ten-year survival trends of neovascular age-related macular degeneration at first presentation. Br J Ophthalmol. 2021;105(12):1688–95. 10.1136/bjophthalmol-2020-317161 33011683

[31] Finger RP, Puth MT, Schmid M, Barthelmes D, Guymer RH, Gillies M (2020). Lifetime Outcomes of anti-vascular endothelial growth factor treatment for neovascular age-related macular degeneration. JAMA Ophthalmol.

[32] Fu DJ, Keenan TD, Faes L, Lim E, Wagner SK, Moraes G (2021). Insights from survival analyses during 12 years of anti-vascular endothelial growth factor therapy for neovascular age-related macular degeneration. JAMA Ophthalmol.

[33] Schmidt-Erfurth U, Kaiser PK, Korobelnik JF, Brown DM, Chong V, Nguyen QD (2014). Intravitreal aflibercept injection for neovascular age-related macular degeneration: ninety-six-week results of the VIEW studies. Ophthalmology.

[34] Eleftheriadou M, Vazquez-Alfageme C, Citu CM, Crosby-Nwaobi R, Sivaprasad S, Hykin P (2017). Long-term outcomes of aflibercept treatment for neovascular age-related macular degeneration in a clinical setting. Am J Ophthalmol.

